# Quality of cervical cancer screening in a Brazilian sample: cross-sectional study

**DOI:** 10.15649/cuidarte.3597

**Published:** 2025-07-22

**Authors:** Liane de Oliveira Serra Tochetto, Maria Meimei Brevidelli, Tainara Rodrigues Santos, Michelle Samora de Almeida, Edvane Birelo Lopes De Domenico

**Affiliations:** 1 Universidade Federal de São Paulo (Unifesp), São Paulo (SP), Brasil. E-mail: lianeserra@gmail.com (Unifesp) São Paulo Brasil lianeserra@gmail.com; 2 Universidade Paulista (Unip), Instituto de Ciências da Saúde, São Paulo (SP), Brasil. E-mail: maria.brevidelli@docente.unip.br (Unip) São Paulo Brasil maria.brevidelli@docente.unip.br; 3 Universidade Federal de São Paulo (Unifesp), São Paulo (SP), Brasil. E-mail: tainara.rodrigues3sants@gmail.com (Unifesp) São Paulo Brasil tainara.rodrigues3sants@gmail.com; 4 Universidade Federal de São Paulo (Unifesp), São Paulo (SP), Brasil. E-mail: michelle.samora@unifesp.br (Unifesp) São Paulo Brasil michelle.samora@unifesp.br; 5 Universidade Federal de São Paulo (Unifesp), São Paulo (SP), Brasil. E-mail: domenico.edvane@unifesp.br (Unifesp) São Paulo Brasil domenico.edvane@unifesp.br

**Keywords:** Uterine Cervical Neoplasms, Papillomaviridae, Papanicolaou Test, Mass Screening, Neoplasias del Cuello Uterino, Papillomaviridae, Prueba de Papanicolaou, Tamizaje Masivo, Neoplasias do Colo do Útero, Papillomaviridae, Teste de Papanicolau, Rastreamento em Massa

## Abstract

**Introduction:**

In Pap test reports, sample quality is assessed based on the presence of squamous-columnar junction (SCJ) cells and atypia.

**Objective:**

To assess the quality of Pap test samples by analyzing the frequency of SCJ cells in Pap test results and examining their association with sociodemographic, clinical, and professional-related variables.

**Materials and Methods:**

A cross-sectional study was conducted using 1.251 reports collected from primary healthcare facilities in São Paulo, SP, Brazil. The chi-square test was used to evaluate potential associations, and the test for equality of two proportions was used to compare responses to the same variable. Odds ratios (ORs) with 95% confidence intervals (CIs) were calculated to determine the strength of associations.

**Results:**

Squamous epithelial cells were the most frequently observed, appearing alone in 50.80% of the samples, while the frequency of SCJ cells was 48.60%, significantly higher among women aged 25-39 years (p<0.001). The presence of inflammation (OR = 7.7; 95% CI: 1.00–50.00), bacilli (OR = 1.8; 95% CI: 1.40–2.20), and cytological atypia (OR = 4.6; 95% CI: 2.00–10.00) was more likely in samples containing SCJ cells. The absence of atrophy, the presence of moderate cytolysis, and red blood cells were significantly associated with the presence of SCJ cells.

**Discussion:**

The study demonstrated that SCJ cells were absent in most samples, which may compromise the quality of cervical cancer screening.

**Conclusion:**

The main indicators of sample quality in Pap test reports fell below recommended standards, particularly among women aged 40-59 years, revealing deficiencies in the quality of cervical cancer screening.

## Resumen

**Introducción:**

En los informes de las pruebas de Papanicolaou, la calidad de las muestras se evalúa con base en la presencia de células de la unión escamocolumnar (UEC) y de atipias.

**Objetivo:**

Evaluar la calidad de las muestras recolectadas para pruebas de Papanicolaou analizando la frecuencia de células de la UEC en los resultados de citologías y examinar su asociación con variables sociodemográficas, clínicas y relacionadas con el profesional.

**Materiales y métodos:**

Este estudio transversal utilizó 1.251 informes recolectados en unidades de atención primaria de salud en São Paulo, SP, Brasil. Se utilizó la prueba de chi-cuadrado para analizar asociaciones y la prueba de igualdad de dos proporciones para comparar respuestas de una misma variable. Las razones de probabilidad con intervalos de confianza (IC) de 95% se usaron para identificar la magnitud de las asociaciones.

**Resultados:**

Las células del epitelio escamoso fueron las más frecuentemente encontradas (50,80%), mientras que la frecuencia de células de la UEC fue del 48,60%, que fue significativamente mayor entre mujeres de 25 a 39 años (p<0,001). La presencia de inflamación (OR= 7,7; IC 95%; 1,00-50,00), bacilos (OR= 1,8; IC 95%: 1,40-2,20) y atipia citológica (OR= 4,6; IC 95%; 2,00-10,00) fue más probable en las muestras que contenían células SCJ. La ausencia de atrofia, citólisis moderada y glóbulos rojos se asociaron con la presencia de células SCJ con significación estadística.

**Discusión:**

El presente estudio demostró que las células SCJ no fueron predominantes en las muestras, lo que compromete la calidad del tamizaje de cáncer de cuello uterino.

**Conclusiones:**

Los indicadores más importantes para determinar la calidad de las muestras en los informes de citología estuvieron por debajo de lo esperado, incluso en el grupo de mujeres de entre 40 y 59 años, lo que evidencia la calidad deficiencias del tamizaje del cáncer cervical.

## Resumo

**Introdução:**

Nos relatórios citopatológicos de exames de Papanicolau, a adequação das amostras é avaliada pela presença de células da junção escamo-colunar (JEC) e pela presença de atipias.

**Objetivo:**

Avaliar a qualidade das amostras coletadas em testes de Papanicolau considerando a frequência de células da JEC nos resultados de citologia e examinar a associação entre a frequência de células da JEC e variáveis sociodemográficas, clínicas e relacionadas aos profissionais.

**Materiais e métodos:**

Foi realizado um estudo transversal utilizando 1.251 laudos coletados em unidades de atenção primária à saúde em São Paulo, SP, Brasil. Testes de qui-quadrado e de igualdade de duas proporções foram utilizados. Razões de chance com IC 95% foram usados para identificar a magnitude das associações.

**Resultados:**

Células do epitélio escamoso foram as mais frequentemente encontradas (50,80%), enquanto a frequência de células da JEC foi de 48,60%, sendo significativamente maior entre mulheres com idades entre 25 e 39 anos (p<0,001). A presença de inflamação, bacilos e atipia citológica foi OR= 7,7 vezes (IC 95%; 1,00-50,00), OR= 1,8 vezes (IC 95%: 1,40-2,20) e OR= 4,6 vezes (IC 95%; 2,00-10,00) mais provável, respectivamente, em amostras contendo células SCJ. A ausência de atrofia, citólise moderada e hemácias foram associadas à presença de células SCJ com significância estatística.

**Discussão:**

O presente estudo demonstrou que as células SCJ não foram predominantes nas amostras, o que compromete a qualidade do rastreamento do câncer cervical.

**Conclusões:**

Os indicadores mais importantes para determinar a adequação das amostras nos relatórios citopatológicos estavam abaixo do esperado, inclusive no grupo de mulheres entre 40 e 59 anos, evidenciando a qualidade insatisfatória do rastreamento do câncer cervical.

## Introduction

In Brazil, the cytology test, commonly known as the Pap test, is the recommended method for cervical cancer screening. Pap test is advised for women aged 25 to 64, as this age range shows a higher incidence of treatable high-grade lesions. This recommendation may also include trans men and nonbinary individuals assigned female at birth^1^,^2^. Effective screening depends on the proper collection of samples, which should include endocervical cells to increase the likelihood of detecting abnormalities such as cervical intraepithelial neoplasia (CIN). The presence of squamous, glandular, and/or metaplastic cells in the collected samples determines whether the test is classified as satisfactory or unsatisfactory. Additionally, the presence of epithelial cells and the absence of cytological abnormalities influence how often a woman should undergo subsequent tests^1^. 

The cervix is the lowermost part of the uterus, and its shape and size can vary depending on a woman’s age, parity, and hormonal status. This part comprises both simple columnar epithelium and squamous epithelium, and the junction between them is known as the squamocolumnar junction (SCJ). The SCJ is a well-defined anatomical landmark, and its location can change based on factors such as age and hormonal status. Within the transformation zone (TZ), the columnar epithelium is replaced by metaplastic cells, making this area most susceptible to human papillomavirus (HPV) infection^1^,^3^,^4^. 

Unsatisfactory specimens in cervical cytopathology reports cannot be appropriately analyzed for various reasons, such as the presence of acellular material in 75% of the smear, blood, pus cells, drying artifacts, external contaminants, or intense cellular overlap. Therefore, Brazilian protocols establish two criteria to ensure the reliability of cervical cytopathology samples: the proportion of unsatisfactory tests reported by the laboratory and the number of slides lacking representative epithelial cells from the transformation zone^1^,^5^. 

The quality of screening is a global concern as cervical cancer remains a serious public health problem. The World Health Organization (WHO), in partnership with other international organizations, including the Union for International Cancer Control (UICC), is working to eliminate cervical cancer by 2030 as part of the Sustainable Development Goals^6^. 

Every year, 56,000 women in Latin America and the Caribbean are diagnosed with cervical cancer, and 28,000 of them do not survive. Most of these women belong to lower socioeconomic strata, a pattern also observed in Brazil. Most of these cases are caused by the human papillomavirus (HPV), particularly in the SCJ^6^,^7^. 

The presence of HPV does not necessarily lead to a diagnosis of cervical cancer; however, it does indicate exposure to risk and the potential for microtrauma and carcinogenesis, especially in younger tissue, where infection and precancerous lesions are more likely to develop^1^,^4^. Overall, this process unfolds over a 10- to 20-year period, making accurate screening essential to reduce the incidence of cases. In addition, public policies that promote HPV vaccination play a critical role in preventing it^8^,^9^. 

Therefore, identifying and addressing deficiencies in the cervical cancer screening process must be an ongoing effort in national cervical cancer prevention and control programs. This study focused on the quality of cervical smear samples, with the objectives of identifying the frequency of SCJ cells in medical reports of cervical smears and verifying the association between the frequency of SCJ cells in Pap test reports and the following variables: age, pregnancy status, inflammation, atrophy or atrophy with inflammation, microbiology, other abnormal cells, and the professional who collected the sample. 

## Materials and Methods

This cross-sectional, quantitative study is based on the analysis of clinical reports from Pap tests performed at 16 Primary Healthcare Units (Unidades Básicas de Saúde [UBS]) that provide free healthcare services to the population of 51 neighborhoods in São Paulo, SP, Brazil. São Paulo City Hall, through the Municipal Health Department, provided data based on the 2018 population projection derived from the 2010 census conducted by the Brazilian Institute of Geography and Statistics. The region has 289,759 inhabitants, of whom 149,481 are women. According to the Brazilian Ministry of Health, 83,006 of these women are between 25 and 64 years old, the group at risk for cervical cancer, with 46.5% relying on the Brazilian Unified Health System (SUS)^10^. 

The study population consists of Pap test reports of women aged 25-64 years who received care at UBSs between August 2018 and January 2019, whether sexually active or not. To control selection bias, all women within this age range whose samples were collected on the scheduled data collection days at the participating units were invited to participate in the study. Researchers were not involved in the interaction between participants and healthcare workers. In addition, researchers did not interfere with the technique used to collect the Pap smears. 

The sample size was calculated considering a quantitative variable and finite population according to the following parameters: a 3% margin of error, an effect size of 0.50, and a 95% CI. A minimum sample size of 1,038 reports was determined. However, considering that it would not incur increased costs or affect the results, we decided to include all the samples collected on the days the interviewer visited the UBS; hence, a larger number of reports was assessed in this study (n=1,251). 

Gynecologic material is collected at the UBSs by physicians and nurses, either through scheduled appointments or daily walk-in services. Eligible women were invited by researchers to participate in the study shortly before collecting the specimen. Once the study authorizations were obtained, participants underwent Pap smear collection by the designated healthcare worker at the UBS. Personal and clinical data were properly recorded in the UBS’s collection logbook, ensuring control for subsequent report verification. 

After testing, the collected material was sent to the laboratory contracted by the São Paulo Municipal Government (Associação Fundo de Incentivo à Pesquisa [AFIP]) for analysis and reporting. Reports were returned to UBS in 20 days or less. Upon receiving the reports, the researchers used a data collection instrument that included the following items: collection site, date of the test, participant's initials and date of birth, pregnancy status, whether the sample was satisfactory, whether the report was available, representative epithelia in the sample (squamous, glandular, metaplastic), microbiology (Cocci bacilli, Candida spp., Gardnerella, Trichomonas), additional information (atrophy, atrophy with inflammation, inflammation), benign changes (specify), cellular atypia (specify), and the professional who performed the test (physician or nurse). 

The prevalence of SCJ cells in cervical cell samples was considered the primary outcome variable. Secondary variables included specimen adequacy, presence of epithelial cells, patient age, pregnancy status, professional responsible for specimen collection (nurse or physician), benign changes observed, and atypia. Reports were scored for sample adequacy and were considered unsatisfactory if they had collection problems such as insufficient cellular material, patient name discrepancies, or mismatched identification data between the requisition form and slide identification. 

Data analyses were organized using Visual Basic for Applications (VBA) software and analyzed with the Statistical Package for the Social Sciences (SPSS), version 20.0. The chi-square test was used to assess potential associations between two or more variables, while the test for equality of two proportions was used to compare responses to the same variable. Odds ratios (ORs) with their corresponding 95% CIs were calculated to determine the strength of associations. If the value 1 fell within the 95%CI, the association was not considered significant. The full data collected is freely accessible and available for consultation on Open Science Framework^11^. 

The Human Research Ethics Committee at Universidade Federal de São Paulo (opinion report No. 0479/2018) and the São Paulo Municipal Health Department (opinion report No. 2.786.369) have approved this study. Women whose reports were selected were asked to sign free and informed consent forms. 

## Results

The samples of most reports (99.40%; n=1,244) were considered satisfactory. Regarding the frequency of epithelial types found in the samples (Table 1), the squamous epithelium alone was found in 50.80% (n=636) of the reports. The presence of two or more epithelial types (squamous-glandular, squamous-metaplastic, squamous-glandular-metaplastic) was found in 48.30% (n=605) of the reports. 

**Table 1:** Representativeness of epithelial cell types found in cytopathological reports

Epithelia	%(n)
None	0.60 (7)
Squamous	50.80 (636)
Glandular	0.20 (3)
Squamous-glandular	29.40 (368)
Squamous-metaplastic	0.60 (8)
Squamous-glandular-metaplastic	18.30(229)

The presence of SCJ cells was 48.60%. The difference compared to their absence (51.40%) was not statistically significant (p=0.162). 

Regarding patient age and the presence or absence of SCJ cells in the specimens (Table 2), a higher frequency of SCJ cells was observed in women aged 25-39 years (56.10%; n=274). Conversely, no SCJ cells were found in 53.60% of women aged 40-59 years and 72.70% of women aged 60-64 years. These differences were statistically significant (p<0.001). 

**Table 2:** Association between SCJ cells and age, pregnancy status, and the professional collecting samples. %(n)

Variables	Total	Absent SCJ	Present SCJ	P-Value
	(1244)	(636)	(608)	
Age group				**<0.001**
25-39	100 (488)	43.80 (214)	56.10 (274)	
40-59	100 (668)	53.60 (358)	46.40 (310)	
60-64	100 (88)	72.79 (64)	27.30 (24)	
Pregnancy				0.259
Yes	1.40 (17)	1.70 (11)	1.00 (6)	
No	98.60 (1227)	98.30 (625)	99.00 (602)	
Professional				**0.022**
Nurse	100 (1073)	52.50 (563)	47.50 (510)	
Physician	100 (51)	51.00 (26)	49.00 (25)	
Not reported	100 (120)	39.20 (47)	60.80 (73)	

SCJ: Squamo-Columnar Junction

Table 2 also shows that there was no association between patient's pregnancy status and the presence of SCJ cells (p=0.259). Pregnant women had the lowest number of tests, which may explain the low number (1.40%; n=17) of reports with SCJ cells among them. Besides, Table 3 shows a statistically significant association between the presence of SCJ cells in the specimens and those collected by nurses (p=0.02). Nearly 10% of the reports did not include information about the professional who collected the specimen.

Table 3 shows the total number of atypia regions in samples with and without SCJ cells and the distribution of these atypia regions by classification. Atypia was observed in only 2.90% of reports (n=36). Cytological changes due to atypia were found in 4.80% of reports with SCJ cells, showing a statistically significant association (p<0.001). The difference of 4.80% versus 1.10% (p<0.001) underscores the importance of adequate Pap smear for cervical cancer screening.

**Table 3:** Association between cytological atypia and the presence or absence of SCJ cells. %(n).

Cytological Atypia	Total	Absent SCJ	Present SCJ	P-Value
	(1244)	(636)	(608)	
ASC-US	2.20 (27)	0.90 (6)	3.50 (21)	**0.002**
ASC-H	0.20 (3)	0.00 (0)	0.50 (3)	**0.039**
AGC-H	0.10 (1)	0.00 (0)	0.20 (1)	0.304
LSIL	0.30 (4)	0.20 (1)	0.50 (3)	0.290
HSIL	0.10 (1)	0.00 (0)	0.20 (1)	0.304
Total	2.90 (36)	1.10 (7)	4.80 (29)	** <0.001**

ASC-US = atypical squamous cells of undetermined significance (possibly non-neoplastic. SC-H = Atypical squamous cells of undermined significance −cannot exclude a high-grade lesion. AGC-H = atypical glandular cells of undetermined significance −cannot exclude a high-grade lesion. LSIL= Low-grade squamous intraepithelial lesions (including HPV cytopathic effect and grade I cervical intraepithelial neoplasia). HSIL = High-grade squamous intraepithelial lesion (comprising grade II and III cervical intraepithelial neoplasia).

Cytological changes observed in the samples with and without SCJ cells deserve attention because they may indicate precancerous conditions. However, Table 3 also shows that the samples without SCJ cells did not show findings of precancerous lesions or changes indicative of malignancy (ASC-H, AGC-H, and HSIL) that were identified only in the samples with the presence of SCJ cells.

Microbiological variables and other abnormal cells were identified but were not significantly associated with the presence or absence of SCJ cells. However, inflammation was present in 99.30% of the reports. Table 4 shows the ORs for some variables in relation to the presence or absence of SCJ cells. 

**Table 4:** Odds ratios (ORs) for association between study variables and the presence or absence of SCJ cells

Variable	OR (95% Cl)	P-Value
Inflammation (Yes)	7.70 (1.00-50.00)	**0.006**
Atrophy (Yes)	6.80 ( 3.80- 12.10)	**<0.001**
Bacilli (Yes)	1.80 (1.40- 2.20)	**0.003**
Scarse Flora	0.00 (0-Inf)	0.976
Total Cytological Atypia (Yes)	4.60 (2-10)	**0.016**
ASC-H (Yes)	1.00 (0-Inf)	0.307
ASC-US (Yes)	3.90 (1.5- 9.1)	0.064
Karyomegaly (Yes)	1.00 (0-Inf)	**0.011**
Moderate Cytolysis (Yes)	2.20 (1.4-3.4)	**<0.001**
Hypoestrogenism (Yes)	2.10 (1.10- 4.09)	**0.001**
Red Blood Cells presence (Yes)	4.60 (2.40- 8.30)	**<0.001**

*OR and/or 95%CI of the OR was not calculated due to division by zero. ASC-US = Atypical squamous cells of undetermined significance (possibly non-neoplastic) . ASC-H = Atypical squamous cells of undetermined significance -cannot exclude a high-grade lesion.

Samples with SCJ cells were 1.8 times more likely to contain bacilli (OR = 1.8; 95% CI: 1.40–2.20) and 4.6 times more likely to exhibit cytological atypia (OR = 4.6; 95% CI: 2.00–10.00). They were also 3.9 times more likely to show ASC-US (OR = 3.9; 95% CI: 1.50–9.10) and 7.7 times more likely to present inflammation (OR = 7.7; 95% CI: 1.00–50.00). Conversely, samples with SCJ cells were 6.8 times more likely to lack atrophy (OR = 6.8; 95% CI: 3.80–12.10; p <0.001), 2.2 times more likely to present moderate cytolysis (OR = 2.2; 95% CI: 1.40–3.40; p <0.001), and 4.6 more likely to show the presence of red blood cells (OR = 4.6; 95% CI: 2.40–8.30; p <0.001). 

## Discussion

The present study has demonstrated that SCJ cells were not predominant in the specimens, compromising the quality of cervical cancer screening. Research has shown that the proportion and presence of SCJ cells can vary, although SCJ cells are a fundamental criterion for an accurate diagnosis^12^,^13^. 

The significant presence of squamous epithelium alone in about half of the samples is a factor of uncertainty, as it may lead to false negative results^13^. Conversely, half of the reports were considered satisfactory due to the presence of two or more epithelial cell types, which increases the accuracy of the information. 

In a similar study evaluating the frequency of SCJ cells in Pap test reports, 68.8% of samples were considered satisfactory for the presence of SCJ cells^14^. However, the authors emphasized the need for further discussion and studies focused on improving the collection technique (ensuring that sufficient material is collected for analysis), smear fixation, processing, and laboratory analysis of cytology material. These improvements are essential to ensure diagnostic accuracy and appropriate treatment to prevent the progression of cytopathologic lesions^14^. In addition, measures are needed to identify, address, and reduce deficiencies in the process, as most factors associated with false-negative results depend on the conditions under which specimens are collected. 

In another study, the highest frequency of SCJ cells was found in younger women between the ages of 25 and 39, although the largest group consisted of women between the ages of 40 and 59^15^. Besides, a lower frequency of SCJ cells was found in women aged 60 years or older^16^. This age group also had a high rate of tissue atrophy, a condition that makes cell sampling more difficult^16^. 

Undetermined cellular abnormalities or atypia, some of which could not exclude high-grade squamous intraepithelial lesions, are consistent with findings reported in other studies^17^. Atypia may progress to more severe changes requiring treatment, especially in the presence of high-risk HPV types (16, 18, and 45), as noted in a recent study^18^. Therefore, different types of atypia were evaluated, highlighting one case of high-grade squamous intraepithelial lesion and four cases of low-grade intraepithelial lesions. Although the number of cases is small in relation to the sampled population, these lesions are key indicators. There is a close relationship between the presence of glandular atypia and preneoplastic and malignant neoplastic cervical lesions, underscoring the importance of the quality of the collected material^19^. 

A lower frequency of pregnant women was found in the reports, and less than half of these cases included SCJ cells, which is the ideal condition for reliable evaluation of these patients. Pregnant women have the same risk of developing precancerous lesions as non-pregnant women. A recent Brazilian study^20^ aimed to compare the prevalence of abnormal cervical smear results between pregnant and non-pregnant women, with and without TZ presentation. The study concluded that the presence of TZ cells was associated with higher rates of abnormal cervical smears and that both groups demonstrated the presence of true precursor lesions. These findings underscore the clinical importance of proper specimen collection, including the presentation of endocervical/metaplastic cells, even in pregnant women^20^. 

In examining the relationship between SCJ findings and the professional who collected the specimen, most were collected by nurses. Nurses often excel at mastering the techniques required to perform screening procedures, and they typically receive specialized training through continuing education programs. However, the current dynamics within the healthcare workforce are characterized by high turnover rates, often driven by the pursuit of better pay and working conditions. This turnover can disrupt training processes and hinder the maintenance of best practices^21^. 

A Brazilian study^22^ aimed at exploring the knowledge, attitudes, and practices of physicians and nurses working in the Estratégia Saúde da Família (ESF) in Juiz de Fora, state of Minas Gerais, regarding cervical cancer control, as recommended by the Brazilian Ministry of Health, revealed concerning data. Among the 170 professionals surveyed, representing 93% of the ESF workers in Juiz de Fora, 39.4% demonstrated adequate knowledge, which was associated with younger age and female sex. The prevalence of appropriate attitudes was 59.5%, and the prevalence of appropriate practices was 77.6%, both associated with a longer time since graduation and familiarity with the Brazilian Ministry of Health's support materials. 

Educational programs implemented to train professionals in Pap smear collection have reported improvements in testing quality indicators, such as proper completion of medical records, frequency of testing, a significant increase in satisfactory samples, and the presence of endocervical cells^23^,^24^. These findings underscore the importance of continuing professional education programs that should be conducted intermittently to reinforce adherence to the national protocol for effective cervical cancer screening. 

The presence of various benign abnormalities, whether or not associated with the presence of SCJ cells, should be carefully considered as they may indicate potential future problems. The association between atrophy and the presence of SCJ cells, as observed in the reports, increases the difficulty of obtaining adequate cell samples. Atrophy is common during the climacteric period; however, this relationship does not appear to follow a consistent pattern when inflammation is also present^24^. 

The absence of a statistical association between inflammation and the presence of SCJ cells confirms that inflammatory processes do not affect the quality of the collected material. Inflammatory processes are generally considered benign factors concerning the presence or absence of SCJ cells. However, samples with more than 98% inflammation, regardless of the presence of SCJ cells, raise concerns about sample integrity and the potential impact on the interpretation of these results. These findings exceed the levels observed in a study conducted in southern Brazil, where inflammation was found in 41.1% of the 1,566 reports analyzed^15^. 

Significant cytological changes were identified in the gynecological tests and analyzed separately. In this study, strong associations were found between atrophy, moderate cytolysis, and hypoestrogenism in samples with the presence of SCJ cells. The number of occurrences of cytolysis determines whether it is considered a normal or abnormal condition. Therefore, this condition indicates changes in the vaginal microbiota and inflammatory processes. This is a relevant concern because it is one of the factors that can lead to false-positive results in the diagnosis of cancer and hypoestrogenism^25^. 

Regarding atypical cells, the total presence of these cells was close to the ideal minimum index recommended by the Brazilian Ministry of Health, which ranges from 3% to 10% for the same site and period. To increase the representativeness in the detection of atypia, criteria such as the proper collection of cell samples and the provision of detailed information on forms, including patient data and age, must be considered^26^. 

Although significant lesions were infrequent in this study (one case of HSIL, three cases of ASC-H, and one case of AGC-H), the accurate identification of precancerous lesions is critical for the prevention of cervical cancer. In addition, these precancerous lesions may progress to more serious changes and must be treated to prevent the development of cervical cancer^17^. 

Significant changes, such as the high-grade squamous intraepithelial lesion (HSIL) identified in one of the reports in this study, could have been identified and treated at earlier tests, resulting in less epithelial disruption. The same is true for the findings of atypical glandular cells of undetermined significance with a possibility of HSIL (AGC-H). In addition, these changes do not develop in a short period; instead, they often progress over a period of up to ten years, suggesting that preexisting conditions could have been treated earlier with more accurate diagnoses^6^,^9^. 

Despite the small number of cases in the study population, these lesions are key indicators, and there is a strong relationship between the presence of glandular atypia and preneoplastic and neoplastic cervical lesions^5^. The findings of this study showed that samples containing SCJ cells were more likely to present cytological atypia and inflammation but without statistical significance. 

Studies with smaller sample sizes have shown a higher incidence of lesions in squamous and glandular cells^5^,^27^. Therefore, the presence of SCJ cells is even more critical to ensure accurate results. In addition, there are specimens with atypia of undetermined significance in squamous and glandular tissues, without excluding the possibility of high-grade lesions. This is an important finding because there is no certainty that such changes will not progress to more serious conditions such as adenocarcinoma. 

The study has several limitations. The first is inherent to its cross-sectional design, which provides a snapshot of a specific period, limiting the ability to observe changes over time. The second limitation relates to the use of a non-probabilistic sampling method, which may not accurately reflect the proportion of tests performed at each participating primary healthcare unit. 

Primary care services are responsible for cervical cancer screening. They should, therefore, use the data from this review to inform and guide discussions on the need for interventions to improve the quality of cervical cancer screening. 

These services should implement protocols that regularly assess specimen adequacy in Pap test reports, with particular attention to the presence of SCJ cells. Evaluating the quality of Pap test reports is critical for healthcare managers to ensure patient safety by taking appropriate action when inaccurate results are observed. For future research, we suggest including variables such as the frequency with which women undergo screening tests and the frequency with which they follow up on their screening test results. 

## Conclusions

 The quality of Pap smear tests was considered unsatisfactory due to the absence of SCJ cells in most Pap test reports, indicating a rate well below the standards established by national protocols. The lower frequency of atypia in the reports, another important quality indicator, reinforces the concern that diagnoses may still be inaccurate despite an adequate sample size.

 The most representative age group was 40 to 59 years. However, the highest frequency of SCJ cells was observed among women between the ages of 25 and 39, a group in which abnormalities are less common. Whether the notable decrease in SCJ cells in reports from pregnant women was due to the small number of samples from this group could not be determined.

 The frequency of SCJ cells was associated with higher frequencies of bacilli, cytological atypia, ASC-US, and the presence of inflammation. Conversely, SCJ cells were significantly associated with the absence of atrophy, the presence of moderate cytolysis, and red blood cells. Although most of the Pap tests were performed by nurses, the proportion of reports with or without the presence of SCJ cells was the same as those performed by physicians.
